# Durability of GFRP and CFRP Bars in the Pore Solution of Calcium Sulfoaluminate Cement Concrete Made with Fresh or Seawater

**DOI:** 10.3390/polym15153306

**Published:** 2023-08-04

**Authors:** Tuanjie Wang, Abdul Ghani Razaqpur, Shaoliang Chen

**Affiliations:** 1 Sino-Canada Joint R&D Centre on Water and Environmental Safety, College of Environmental Science and Engineering, Nankai University, Tianjin 300350, China; wtj_lts@mail.nankai.edu.cn (T.W.); chenshaoliang@mail.nankai.edu.cn (S.C.); 2 College of Civil Engineering, Zhengzhou University of Industrial Technology, Zhengzhou 451150, China; 3 Department of Civil Engineering, McMaster University, Hamilton, ON L8S 4L7, Canada

**Keywords:** calcium sulfoaluminate cement, durability, FRP bar, seawater, multiple mechanical parameters

## Abstract

Calcium sulfoaluminate cement concrete (CSAC) reinforced by fiber-reinforced polymer (FRP) bars, termed bars for brevity, is a good alternative to steel-reinforced concrete in marine environments due to the corrosion resistance of FRP and the lower pH of CSAC. For the first time, multi-mechanical tests are conducted to compare the durability of glass FRP (GFRP) to that of carbon FRP (CFRP) after exposure to CSAC pore solution. The bars were immersed in a simulated pore solution of CSAC made with either fresh water and river sand or with seawater and sea sand. Solution temperature was held constant at 30 °C, 45 °C or 60 °C for 30, 60, 90 and 180 days of immersion. Tensile, horizontal and transverse shear tests, as well as detailed microstructural analyses, were conducted to determine the level and mechanisms of degradation for each type of bar. Sea salt increases the degradation of both bars, but it degrades GFRP more than CFRP. The bars’ retained tensile strength is a reliable indicator of their durability, while their post-exposure horizontal and transverse shear strengths are found inconsistent and counter intuitive. In the GFRP, the fiber, the epoxy matrix and their interface suffered damage, but in the CFRP, the carbon fiber was not damaged. Under the test conditions in this study, the maximum reduction in the tensile strength of the GFRP was 56.9% while that of CFRP was 15.1%. Based on the relevant ASTM standard, the CFRP bar satisfies the alkaline resistance requirement of the standard in the CSAC pore solution with and without salt, whereas the GFRP bar does not meet the same requirement in the above pore solution with salt.

## 1. Introduction

Fiber-reinforced polymer (FRP) bars are characterized by low density, high strength and good corrosion resistance. They are increasingly used as replacement of steel bars in concrete structures exposed to corrosion inducing agents. The durability of FRP bars exposed to sea/tap/deionized water and the alkaline environment of Portland cement (PC) concrete (PCC) has been extensively and systematically investigated over the past two decades [1,2,3,4,5,6,7,8,9,10]. One of the characteristics of PCC is its high pH value, but experiments have shown that FRP, especially glass FRP (GFRP) and basalt FRP (BFRP), are more resistant to degradation in a low-pH environment. The latter is demonstrated by their relatively higher retained horizontal shear [4,5], tensile [3,6,7,8] and flexural [8] strengths. 

Methods to reduce the pH of concrete include addition of pozzolanic materials [3] to PC or replacement of PC by calcium sulfoaluminate cement (CSA) [7] due to the normally lower pH of CSA concrete (CSAC) [7,11,12,13,14,15,16]. CSA has been the subject of large-scale development in China since the beginning of the 1980s [17]. Compared to PC, the CO_2_ emission of CSA is much smaller due to the lower CO_2_ release of its constituent materials and its lower energy consumption during production [13,18,19]. Due to its dense pore structure [20,21] and high resistance to sulfate attack [17,21,22,23], CSA is more suitable for application in marine and other similar aggressive environments. Research has shown [7] that FRP has better durability in a CSA environment. The last study involved the testing of a GFRP bar embedded in mortars made of CSA, PC and a blended CSA-PC cement. It was demonstrated, based on the retained tensile strength of the bar, that the tested bar had the best durability in the low-pH environment of CSA. The matrix or resin of the GFRP bar tested in the last study was vinyl ester.

Holistic evaluation of the durability of a reinforcing bar in an environment requires determination of the deleterious effects of that environment on the bar’s key mechanical properties. These include its tensile, transverse shear and bond strengths. In addition, for quality control, determination of horizontal shear strength may be also necessary. Extensive investigations have been conducted to examine the degradation of GFRP and BFRP bars, and to a much lesser extent, CFRP bars, when exposed to the PC environment [24,25,26,27,28,29,30,31,32,33,34]. It has been reported that, after exposure to PCC simulated pore solution, the deterioration rate among the aforementioned mechanical properties may differ significantly [35]. In [36], it was reported that the transverse shear strength reduction correlated well with the reduction in tensile strength. Several research works [4,5,37,38,39,40,41,42] have used the degradation of horizontal/interlaminar shear strength as the sole indicator of the FRP bar durability. Some researchers have indicated that degradation of horizontal shear strength can be correlated to the degradation of the bond between the FRP bar and concrete [35,43,44]. 

While research about FRP durability in PCC made with fresh water and clean sand or with seawater (SW) and sea sand is extensive, similar research on the durability of FRP bars exposed to CSAC and SW-CSAC is scant. Assessment of the durability of FRP in the latter type of concrete based on multiple mechanical strength criteria is particularly lacking. 

The extent and nature of FRP degradation is affected by the chemical composition of the solution to which it is exposed. Since the chemical composition of CSAC pore solution is different from that of the PCC pore solution [12,14,15,18], it is necessary to assess the durability of FRP bars in CSAC concrete made with fresh water and clean sand or with seawater and sea sand. Due to the presence of sea water and sea sand [45,46] in offshore and near-shore regions, concrete structures in these regions could be made more economical and sustainable [47] by using seawater and sea sand.

Considering the above discussion and the paucity of research on FRP durability in CSA concrete, this study, for the first time, comprehensively investigates and compares the durability of GFRP and CFRP bars, made with an epoxy matrix in CSAC simulated pore solution. The bars are immersed in solutions whose chemical composition is designed to simulate the pore solutions of CSAC made with clean sand and fresh water or with sea sand and seawater. The solution temperature is held constant at 30 °C, 45 °C or 60 °C for 30, 60, 90 and 180 days. For design purposes, a reinforcing FRP bar must not only have a certain guaranteed tensile strength, it must also possess minimum transverse and interlaminar shear strength. Therefore, appropriate tests are conducted in accordance with the relevant ASTM standards [48,49,50,51] to determine the effects of exposure temperature, duration and type of solution on each of the foregoing strengths. Energy-Dispersive X-ray Spectroscopy (EDS)-mapping and EDS-point scanning are applied to investigate the diffusion of ions and the degradation mechanisms of the bars while Fourier Transform Infrared (FTIR) spectroscopy is used to explore the degradation of epoxy.

The present research is focused on the durability of GFRP and CFRP bars made with epoxy matrix. The bars are immersed in CSAC simulated pore solution, with and without sea salt. In more detail, it aims to establish the extent and mechanisms of these bars’ degradation as a function of exposure temperature and duration. The study will elucidate whether the tested bars, when used as reinforcement in CSAC, can satisfy the ASTM D7957/7957M-22 Standard [52] requirements for alkali resistance. Since CSAC is believed to offer certain environmental and durability advantages over conventional Portland cement concrete in the marine environment, the study also investigates the effects of sea salt on the durability of these bars.

## 2. Materials and Methods

### 2.1. Materials

**Figure 1:**
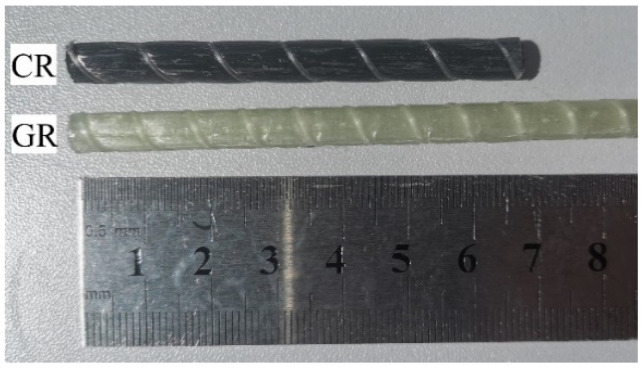
Typical GFRP and CFRP bars samples investigated.

As shown in Figure 1, GFRP and CFRP bars composed of E-glass/carbon fibers and epoxy resin were acquired from the same manufacturer in China. The bars were manufactured using the pultrusion method [53]. The detailed physical and mechanical properties of unexposed or reference GFRP(GR) and CFRP(CR) bars, determined in the current study based on the relevant ASTM standards, are shown in Table 1. Note, each row of column 3 of the table refers to the relevant ASTM standard for determining the companion physical or mechanical property, which is specified in column 2 of the same table. 

### 2.2. Immersion Solution Chemistry

The GFRP and CFRP bars were immersed in simulated CSAC pore solutions, termed PS and SS. Based on data in [11,12], PS had the chemical composition of the pore solution of a CSAC made with fresh water and cleaned sand, while SS had that of a CSAC made with seawater and sea sand. The PS principal chemical components, as shown in Table 2, were obtained in [11] by analyzing the pore solution of CSAC. The simulated seawater chemical composition in Table 2 is specified by ASTM D1141-98 [57]. As can be noticed in Table 2, the SS contains the same chemicals as PS plus the chemicals in simulated sea water. The two solutions have the same pH value. All chemical materials, except potassium hydroxide, were analytical pure, while the purity of potassium hydroxide was greater than 85%.

### 2.3. Test Setup

**Figure 2:**
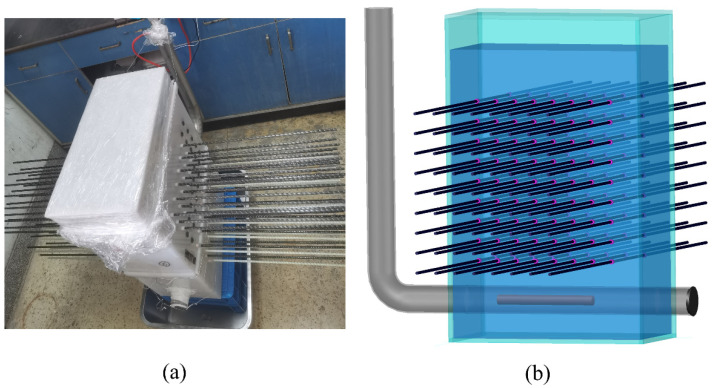
Immersion test setup (**a**) photograph and (**b**) schematic diagram.

The setup for conditioning the FRP bars, henceforth referred to as bars for brevity, in the solutions is illustrated in Figure 2a,b. In total, six identical setups were used. A typical setup involved a rectangular plastic tank filled with one of the solutions. Holes were drilled in the two opposite walls of the tank to pass the 1000 mm long bar samples through the tank, and then the holes were sealed with silicon and water-resistant tape. The width of the tank was 200 mm, which is equal to the length of the conditioned part of each bar sample. Thus, as shown in Figure 2, approximately 400 mm long segments of the bar projected from two walls of the tank. An L-shaped stainless-steel pipe, Figure 2b, was placed inside the tank and a heating element was inserted into the pipe. The pipe was filled with water that was heated by the heating element. The solution temperature was maintained constant via a thermostat with a precision of ±1 °C, and the water level in the pipe was controlled via an automatic controller. 

In the current investigation, the bars were conditioned under constant temperatures of 30, 45 and 60 °C for periods of 30, 60, 90 and 180 days. The immersion durations and the highest immersion temperature in this study are based on the ASTM D7705/D7705M-12 [48] recommendation. It states that specimens for procedure A, the one applied in this study, shall be immersed in the alkaline solution at 60 ± 3 °C (140 ± 5 °F) for exposure times of 1, 2,3 or 6 months, unless longer exposure periods are specified. The temperature of 30 °C in this study is selected mainly based on the annual average surface temperature of seawater in the South China Sea [58]. Since the functional relationship between the long-term durability of FRP bars as a dependent variable, and the immersion temperature and duration as independent variables requires a minimum of three values [59] for each independent variable, the 45 °C temperature, which lies midway between 30 and 60 °C, was chosen as the third immersion temperature. It is important to note that three similar temperatures were also used in [6,59,60,61,62] to investigate the durability of FRP bars in conventional concrete pore solution.

Companion virgin GFRP and CFRP bars were tested as references and were designated as GR and CR, respectively. For easy reference, the conditioned bars are designated as BTSTT#D#, where BT = bar type = C for carbon and G for glass, ST = solution type = PS or SS, T# = conditioned solution temperature in °C, e.g., T30, D# = the length of the conditioning period in days, e.g., D60. For example, CSST60D90 represents the group of CFRP bars immersed in the SS solution under constant 60 °C for 90 days.

### 2.4. Mechanical Tests

**Figure 3:**
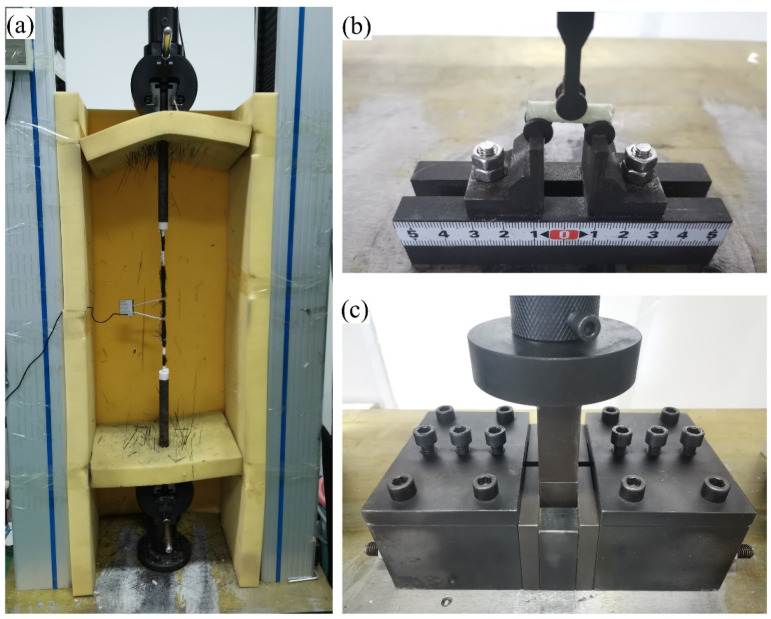
Test rigs (**a**) tensile, (**b**) horizontal shear, (**c**) transverse shear.

The rigs for the tensile, horizontal and transverse shear tests of the bars are shown in Figure 3a–c, respectively. To determine the tensile and horizontal shear strengths, six replicate specimens were tested in each case, while for transverse shear, five specimens were tested. 

#### 2.4.1. Tensile Test

The tensile test was conducted in accordance with ASTM D7205/D7205M-21 [51] specifications using a 100 kN universal testing machine. Steel tubes that were 300 mm long were used to anchor the bar ends, and the tubes were grouted with a mixture of silica sand and epoxy. The bar free length between the anchors was 400 mm, with the middle 200 mm being the conditioned part. The bar extension was measured with an extensometer with gauge length of 50 mm. 

#### 2.4.2. Horizontal Shear Test

The horizontal shear test was conducted based on ASTM D4475-21 [49] using the rig in Figure 3b, which complies with the requirements of ASTM D4475-21 [49]. The span-to-diameter ratio was 3 for conditioned bars; the basis for the selected ratio will be explained later. Before the test, the diameter of each specimen was measured at its midspan using digital calipers with 0.02 mm accuracy. Using the same universal testing machine that was used for the tension test, each specimen was loaded at a rate of 1.3 mm/min until shear failure occurred. Horizontal shear strength was calculated per ASTM D4475-21 [49] as

(1) $$S=\frac{0.849P}{d^{2}}$$

where *S* is the horizontal shear strength in Pascal (N/m^2^), *P* is the applied breaking load in N, and *d* is the bar diameter in m.

#### 2.4.3. Transverse Shear Test

The transverse shear test was conducted in compliance with ASTM D7617/D7617M-11 [50] specifications using the rig in Figure 3c. The cross-sectional area was measured as per ASTM D7205/D7205M-21 [51]. Specimens were loaded at a rate of 1.0 mm/min based on the machine crosshead movement. The transverse shear strength was calculated per ASTM D7617/D7617M-11 [50] using Equation (2).

(2) $$\tau_{U}=\frac{P_{S}}{2A}$$

where $\tau_{U}$ is the transverse shear strength in MPa, $P_{S}$ is the maximum or failure force in N, and *A* is the bar cross-sectional area in mm^2^.

### 2.5. Microscopic Analyses

To obtain the bars deterioration evolution and mechanisms after exposure, scanning electron microscopy (SEM) and EDS analyses were conducted. SEM examination was conducted to track the reference and conditioned bars microstructural changes. EDS analysis was performed to obtain the change in the bars’ chemical composition. To avoid damage to the bar surface, each bar was coated with a layer of epoxy before SEM examination. After the epoxy hardened, the bar was cut into small discs. One face of each disc sample was polished with the help of silicon carbide (SIC) papers with grit number ranging between 180 and 10,000. The SEM and EDS examinations were conducted using a TESCAN MIRA LMS scanning electron microscope by TESCAN in Brno, Czech Republic. 

Fourier transform infrared (FTIR) spectroscopy analysis was used to reveal functional group changes in FRP bars components [2]. Powder and cylindrical samples were used for FTIR analysis using the Thermo Scientific Nicolet iS20 spectrometer by Thermo Scientific in Waltham, MA, USA. Powder samples were obtained through sawing the bars and collecting the dust. The saw dust was ground to a very fine powder before the test. The powder was mixed with potassium bromide by grinding them together using a mortar and pestle. Finally, the blended powder was shaped into tablets for FTIR examination. In addition, cylinder samples were prepared by sawing the bars, and the test surface of each specimen was polished using silicon carbide (SIC) papers. All FTIR measurements were conducted using wave numbers from 4000 to 400 cm^−1^, and 64 scans were performed with spectral resolution of 4 cm^−1^.

## 3. Results and Discussion

### 3.1. Visual Observations

#### 3.1.1. Bar Surface Morphology

**Figure 4:**
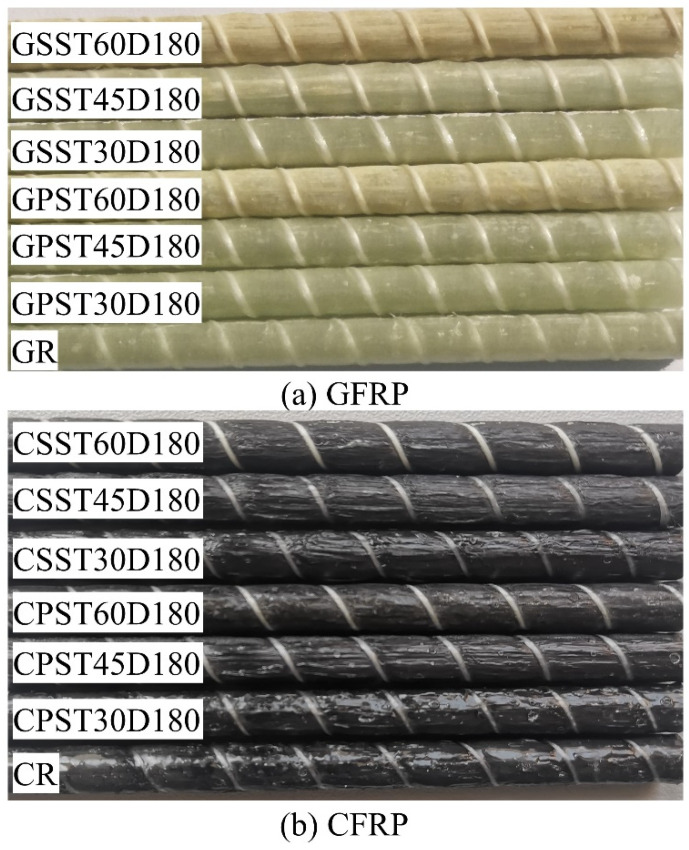
Typical color changes of FRP bars after 180 days of immersion in PS and SS solutions at different temperatures (**a**) GFRP and (**b**) CFRP.

Typical images of reference and conditioned GFRP and CFRP bars after 180 days of exposure are shown in Figure 4a,b, respectively. One can observe that, after 180 days of immersion, the GFRP bars conditioned at 60 °C in both solutions exhibit noticeable change in color from pale green to dull yellow, but those conditioned at 30 and 45 °C show practically no change. The CFRP bars, irrespective of the solution type or temperature, do not exhibit any obvious color change. 

The color change per se cannot indicate significant change in the bar’s mechanical and chemical properties as it may be restricted to the resin on the bar surface. The change may also depend on the type of pigment used to provide color to the bar. 

#### 3.1.2. Tensile Failure Mode

**Figure 5:**
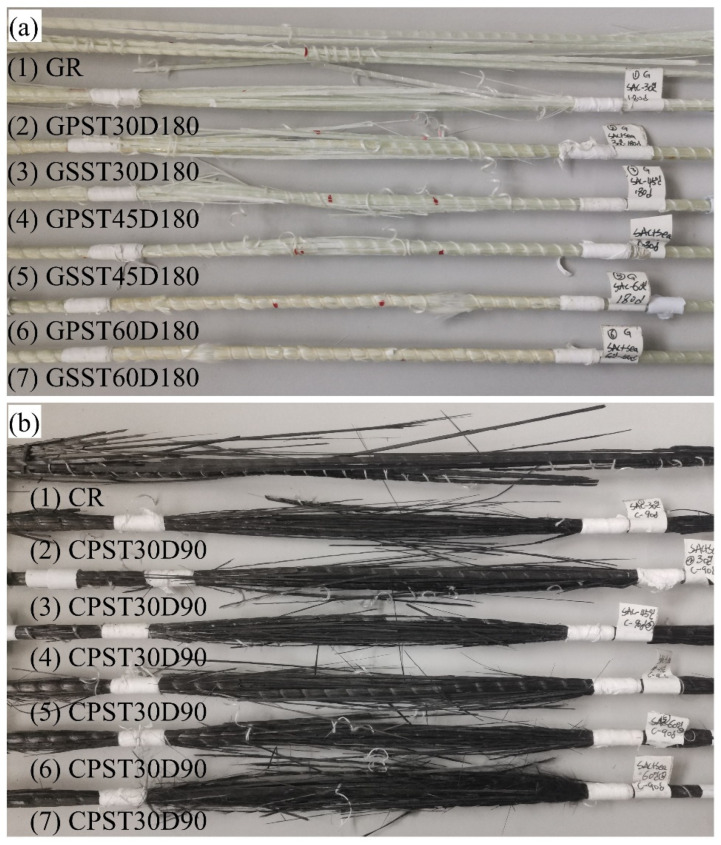
Global versus local tensile failure of conditioned GFRP and CFRP bars (**a**) GFRP and (**b**) CFRP.

Figure 5a,b, respectively, shows typical failed GFRP and CFRP bars after the tensile test. The label on each bar indicates its relevant exposure conditions. With reference to Figure 5, the bars exhibited different failure modes. The reference bars, irrespective of fiber type, experienced fiber rupture or interlaminar shear failure outside the anchors. The conditioned CFRP bars failed similarly. This global failure spanned the entire 200 mm conditioned length. The conditioned GFRP bars, however, had a different type of failure. As Figure 5a shows, with the increase in the immersion temperature, the ruptured section became shorter and localized. The local failure could be indicative of the effect of high temperature [2] on the rapid degradation of glass fibers exposed to the solution. Due to defective sizing, flawed fiber-matrix interface, or nonuniform distribution of voids in the matrix, the fibers in the vicinity of the defects would be more readily accessible to the solution. Since glass fibers are susceptible to attack by alkaline/saline solution [63,64], they would suffer more degradation and early rupture under tension. On the contrary, carbon fibers are immune to attack by the chemicals present in the conditioning solutions in the current study. 

### 3.2. Retained Tensile Strength and Elastic Modulus of the Conditioned Bars

Three hundred GFRP and CFRP bars were tested under tension. Their tensile strength and elastic modulus values were determined as specified in ASTM D7205/D7205M-21 [51] and are reported in Table 3 and Table 4, respectively. In each case, the reported mean strength value and the associated coefficient of variation (COV) are based on data from at least five replicate specimens. In a few cases, the statistical Q-test [65] was used to reject an outlier. 

**Figure 6:**
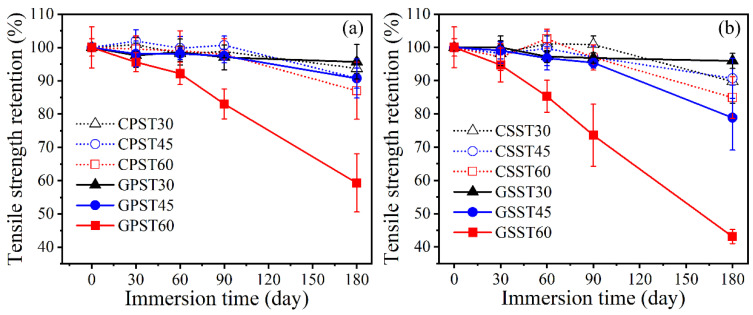
Tensile strength retention of CFRP and GFRP bars after exposure to solution: (**a**) PS (**b**) SS.

Before discussing the data in the last tables, Figure 6a,b show the %retained tensile strength of the bars after immersion in solution PS and SS, respectively. 

As can be noticed, irrespective of the solution type, if the exposure temperature is below 45 °C, neither type of bar exhibits more than 5% reduction in tensile strength after up to 90 days of immersion. Between 90 and 180 days, the rate of deterioration increases, and the SS solution inflicts greater damage on both types of bar than the PS solution does. However, the GFRP consistently suffers higher damage than the CFRP. When the exposure temperature is increased to 60 °C, the GFRP experienced dramatic reduction in strength, resulting in %retained tensile strength of only 59.3% and 43.1% after 180 days of immersion in the PS and SS, respectively. The companion CFRP bar retained approximately 90% of its tensile strength under the same conditions. Although SS inflicted slightly higher damage on CFRP than PS did, the difference is relatively small in the context of the current test conditions. On the contrary, the damage caused to the GFRP by the 60 °C SS was appreciably higher than that caused by the companion PS solution. Since the two types of bars are made by the same manufacturer using the same type of epoxy matrix, it can be argued that the glass fiber is susceptible to major damage in CSA concrete under high temperature (≥60 °C) and prolonged exposure scenarios. 

As carbon fiber is immune from attack by many chemicals, the observed damage to the CFRP bar can be attributed to the degradation of the epoxy matrix and/or the fiber-matrix interface. Based on the relevant ASTM D7957/7957M-22 standard [52], the tested CFRP bar satisfies the alkali resistance requirement of the standard in both the PS and SS solution, whereas the GFRP bar satisfies the same requirement in the PS solution but not in the SS solution.

In design, another important property of any reinforcement is its elastic modulus. A severe reduction in the FRP bar elastic modulus would increase the deflection and crack width of FRP-reinforced concrete structures under applied loads. In the current study, none of the exposure conditions had a practically significant effect on the elastic modulus of the GFRP bar, but the CFRP bar elastic modulus exhibited approximately 8% reduction after 180 days of immersion. Considering the differences between the GFRP and CFRP bars’ fibers diameters, elastic moduli and volumetric fiber ratios, it can be estimated that, under equal tensile load, the interfacial shear stress in the current CFRP bar would be at least 50% higher than that in the companion GFRP bar. Consequently, the likelihood of interfacial damage at the same tensile force level in the CFRP would be higher than that in GFRP. This may explain the higher reduction in the CFRP bar elastic modulus.

#### Statistical Analysis of Tensile Strength

The following analyses were performed using appropriate statistical procedures [66]. The tensile strength data were analyzed to compare the significance of the differences at the 95% confidence interval. The Shapiro–Wilk test [67], generally used to check normality of a sample size of less than 50 [68], was applied to check the normality of the tensile strength data, and the Levene’s test, a standard test for homogeneity of variance [69], was conducted to measure the homogeneity or equality of variances. The appropriate *p*-value was selected when the null hypothesis, or the alternative hypothesis, regarding the equality of variances was tested. The one-way analysis of variance (ANOVA) was used to determine whether there were any significant differences among at least three levels. The least significant difference (LSD) method was used to conduct post-mortem comparison. For example, the influence of immersion time or exposure temperature was mainly used in one-way ANOVA methods.

All samples used for one-way ANOVA satisfied the normality and homogeneity of variance tests. The Independent sample *t*-test was used to determine whether there was any significant difference between two levels. When the data did not satisfy the equality of variances, or when they only contained two levels, an independent sample *t*-test was performed. For both the independent sample *t*-test and one-way ANOVA, a significance level α = 0.05 was selected. Consequently, in this analysis, any *p*-value < 0.05 is considered to reflect significant influence.

Based on the Shapiro–Wilk test results, all the GFRP and CFRP samples satisfied the normality condition, except for GPST60D90 and GSST45D90, so the latter two samples were not used in the one-way ANOVA and the independent sample *t*-test. For assessing the influence of SS versus PS, partial results of the analysis are shown in Table 5. Only the samples with *p*-value < 0.05, that is, those exhibiting the significant influence of the solution type on their retained tensile strength are listed. For constant solution temperature of 30 °C, the results indicate that the addition of sea salt to the pure CSAC pore solution has no significant effect on the retained tensile strength of either type of bar. As far as the CFRP bar is concerned, solution type has no significant effect on its retained tensile strength, regardless of the length of immersion time.

The influence of temperature on retained tensile strength is shown in Table 6. It can be noticed that, for immersion times of 60 days or longer, the GFRP bars immersed in the 60 °C solutions generally exhibit significant differences from those immersed in the companion 30 °C and 45 °C solutions. On the contrary, for immersion periods of less than 90 days, no significant difference is observed between the samples immersed in the 30 °C and 45 °C solutions. As for the CFRP bar, temperature has no significant effect on its retained tensile strength, regardless of the immersion time length. 

The effect of immersion time on retained tensile strength is shown in Table 7. With reference to the last table, for either solution maintained at 30 °C, the length of the immersion time has no significant effect on the GFRP bar retained strength. For GPST45, GPST60 and GSST45, only 180 days of immersion has a significant effect. For GSST60, the interval between any of the two consecutive immersion durations shows significant effect. In the case of the CFRP bar, 180 days of immersion shows significant difference with the other immersion times. However, CSST60 exhibits significant difference between 30 and 60 days and between 60 and 90 days.

Hence, for the current bars, immersion time, especially 180 days, has a significant effect on the retained tensile strength, while temperature and solution type have significantly more effect on the GFRP bars than the CFRP bars.

### 3.3. Horizontal Shear Test Results

#### 3.3.1. Failure Morphology

**Figure 7:**
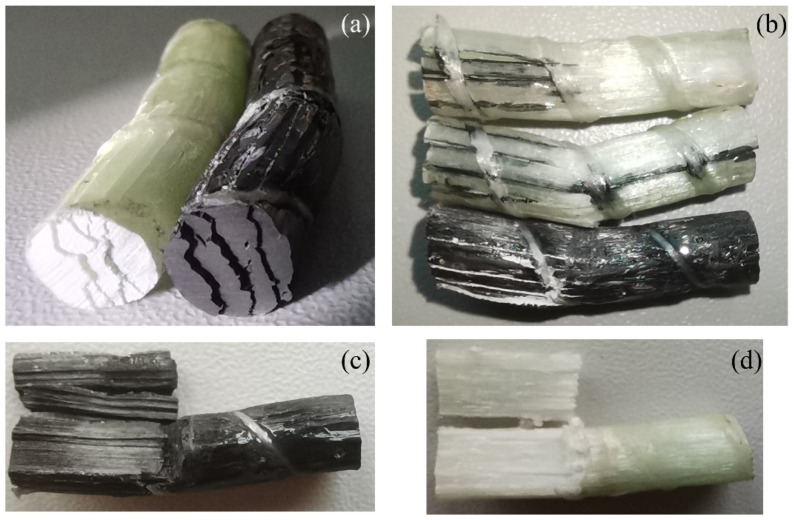
Failure morphology of horizontal shear: (**a**) cross section, (**b**) longitudinal directory, (**c**) vertical section of CFRP bar, (**d**) vertical section of GFRP bar.

Figure 7 shows the failure morphology of the GFRP and CFRP bars after the horizontal shear test. As the applied load was continuously increased, longitudinal cracks suddenly appeared, and the load started to decline. Crack formation was accompanied by a loud sound and the release of some epoxy powder. The cracks formed one or more delaminated planes near and parallel to the neutral plane of the bar cross-section. With further increase of the applied vertical displacement, beyond that corresponding to the peak load, more horizontal failure planes formed as illustrated in Figure 7. Eventually, the part of the bar below the lowest plane fractured in the vicinity of the midspan of the specimen. In most specimens, the failure planes formed asymmetrically on only one side of the externally applied load. This horizontal shear failure morphology is similar to that reported by other researchers [26,32,38,41,44,70]. 

#### 3.3.2. Influence of Span-to-Diameter Ratio on Horizontal Shear Failure

For determining the horizontal shear strength of FRP bars, ASTM D4475-21 [49] suggests testing bar samples with span/diameter ratio not less than 3 nor greater than 6. For a bar of circular cross-section subjected to a pure shear force *F*, the horizontal shear stress *τ* acting on any plane located at distance *h* from the neutral axis can be calculated using Equation (3) [71].

(3) $$\tau =\frac{4}{3}\frac{F}{\pi r^{4}}\left(r^{2}-h^{2}\right)$$

Notice that the maximum shear stress occurs at *h =* 0.

**Figure 8:**
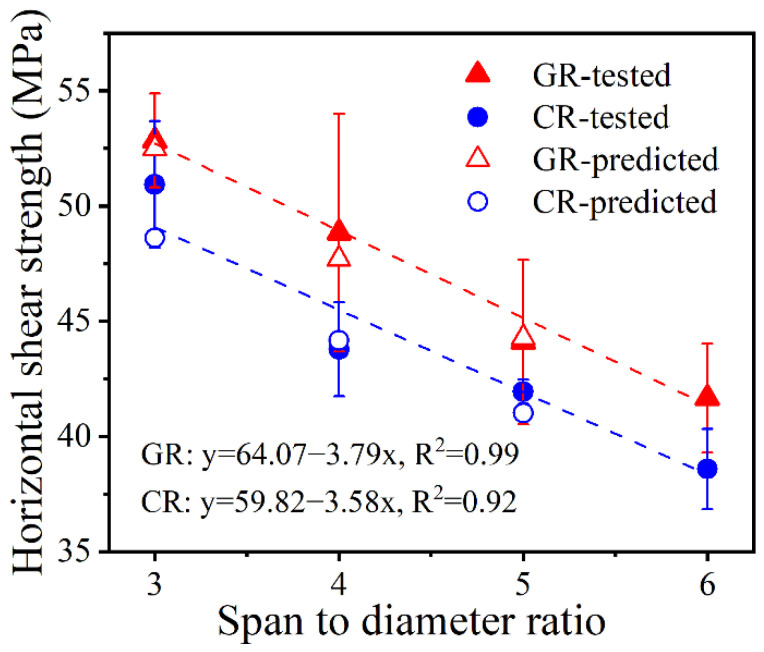
Horizontal shear strength of GR and CR specimens with different span to diameter ratios.

As this standard does not give guidance regarding the selection of a specific ratio, to assess the sensitivity of the current bars horizontal shear strength to this parameter, unconditioned specimens with span-to-diameter ratios of 3, 4, 5 and 6, were tested. The results are shown in Figure 8. 

As Figure 8 shows, within the above *s*/*d* range, the horizontal shear strength decreases almost linearly with the increase in the span-to-diameter ratio. In past works, a similar trend was reported for FRP bars [5,70] and strips [72]. 

Although horizontal shear test on FRP bars has been conducted by several investigators, only a few have analyzed the influence of *s*/*d*. ASTM D4475-21 [49] states that experiments indicate that the horizontal shear strength is a function of the specimen span-to-diameter ratio in most materials. In [70], this parameter was dealt with in detail, and a correction factor was introduced to account for it. According to [70], by knowing the horizontal shear strength of a given bar, *τ_H_*, for any for any *s*/*d* (3 ≤ *s*/*d* ≤ 6), and using it as the reference strength, the corresponding strength other *s*/*d* value can be computed as

(4) $$\tau_{H,S}=c\;\tau_{H,R}$$

(5) $$c=\sqrt[{3}]{\frac{S_{R}}{S}}$$

where *τ_H,S_* is the predicted horizontal shear strength; *c* is the correction factor; *τ_H,R_ is* the reference horizontal shear strength; and *S* and *S_R_* are the target and reference specimen span, respectively.

For the present test specimens, using the measured horizontal shear strength for *s*/*d* = 6 as the reference, the predicted horizontal shear strength for *s*/*d* values of 3, 4 and 5 are computed and plotted in Figure 8. The figure also shows the linear fit to the experimental data and the corresponding R^2^ values. It appears that, for *s*/*d* values between 3 and 6, the horizontal shear strength varies almost linearly, but Equation (4) has the advantage that it may be applicable to even larger *s*/*d* values. Also, it requires horizontal shear strength results for a single *s*/*d* value to be able to predict the corresponding strength for any other *s*/*d* within the above range. 

#### 3.3.3. Horizontal Shear Retention

**Figure 9:**
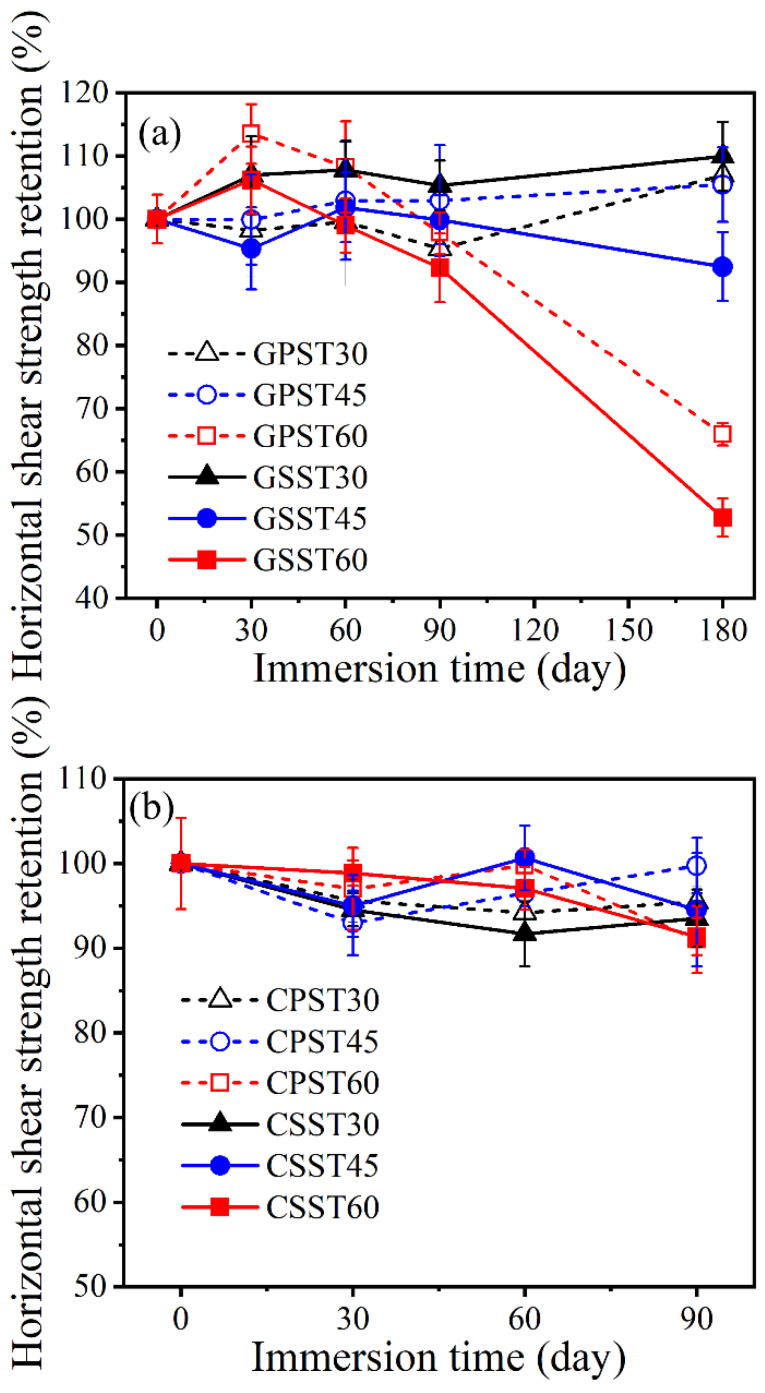
Horizontal shear strength retention (**a**) GFRP and (**b**) CFRP bars.

As mentioned in Section 2.4, the conditioned GFRP and CFRP bars were cut into short test pieces for the purpose of finding the bars’ horizontal shear strength. For each exposure condition, six replicates with *s*/*d* = 3 were tested. Figure 9a,b shows the retained horizontal shear strength of the conditioned GFRP and CFRP bars, respectively.

Based on Figure 9a, under exposure temperatures of 45 and 60 °C, the SS reduced the retained horizontal shear strength of GFRP more than the PS. On the other hand, exposure up to 60 days generally increased the retained shear strength. When it decreased, the reduction was less than 5%. The largest reduction (≈50%) was experienced in the SS solution after 180 days of immersion at 60 °C. The reduction caused by the PS solution under the same conditions was around 35%. The latter levels of damage are both drastic and are in the same ballpark as the tensile strength reduction experienced by this bar under the same exposure conditions. As both the tensile stress and the horizontal shear stress transfer takes place through the fiber-matrix interface, the high level of degradation in the two strengths can be ascribed to the appreciable degradation of the fiber-matrix interface. On the other hand, the tensile strength is also highly dependent on the fiber strength while the horizontal shear strength is not [35,73]. Consequently, the tensile strength degradation is the consequence of the damage incurred by the fibers and their interfaces.

For the CFRP bar, Figure 9b shows the horizontal shear strength degradation for up to 90 days of immersion in the two solutions. The results beyond 90 days are not available because there was an insufficient number of this type of bar to test. Still, the provided data is useful for comparing the extent of damage to the CFRP relative to the GFRP under the same conditions and for the same immersion duration. Figure 9b shows the retained horizontal shear strength fluctuating with increasing exposure time. This type of fluctuation has been also reported in [34]. It is most likely due to the random variations in the mechanical and microstructural properties of the bar along its length rather than some intrinsic material property. Some of the results seem counter intuitive. For example, the samples immersed in the 30 °C solution exhibit more degradation than the ones immersed in the 60 °C solution. As both the GFRP and CFRP bars exhibit similar fluctuations, it confirms that the fluctuation is not caused by intrinsic material property. 

It should be pointed out that, whereas the tensile test shows the strength of the weakest section along the relatively long free length of the bar, a horizontal shear test reflects the shear strength of the weakest plane of a short segment of the bar or of the plane subjected to combined maximum moment and shear. Unlike the tensile strength, which is not a function of the location of the applied tensile load relative to the position of the weakest plane along the bar, the horizontal shear strength is sensitive to the difference between the moment acting on the weakest plane and the maximum moment acting on the test specimen. Increase in the maximum moment to shear ratio may cause failure at a section other than the weakest section along the shear span.

### 3.4. Transverse Shear Strength Results

#### 3.4.1. Influence of Specimen Length

Based on ASTM D7617/D7617M-11 [50], for testing the transverse shear strength of a FRP bar, the length of the test specimen shall be 225 mm even though, theoretically, this type of failure is independent of the specimen length. To investigate the influence of specimen length on the transverse shear strength of the bars in this study, 225, 150 and 100 mm long specimens were tested. At least 8 replicates specimens were tested in each case. The results were statistically analyzed to gauge the influence of the specimen length. 

The one-way analysis of variance (ANOVA) was applied to determine whether there was any significant difference among the 225, 150 and 100 mm levels. As Table 8 shows, all *p*-values of Shapiro–Wilk and Levene’s test are larger than 0.05, which signifies that all the data satisfied the normality test and the equality of variances. Also, the *p*-value of one-way ANOVA for each bar is larger than 0.05, which signifies, as theoretically expected, that the specimen length has no significant influence on its transverse shear strength. 

#### 3.4.2. Transverse Shear Strength Retention

**Figure 10:**
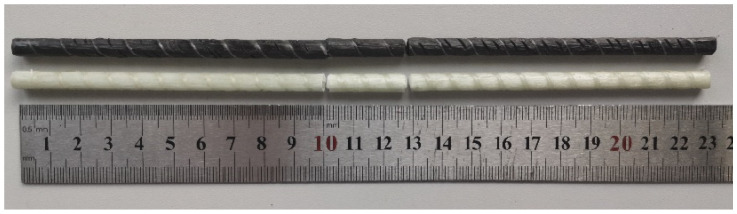
Failure morphology of transverse shear.

Guided by the preceding statistical analysis, the length of the conditioned GFRP and CFRP specimens for the transverse shear test was selected as 100 mm, and five replicate specimens were tested in each case. Figure 10 shows typical CFRP and GFRP samples after transverse shear failure. Regardless of the conditioning environment or the exposure duration, the failure pattern for all the specimens was identical. It was characterized by fractured planes perpendicular to the longitudinal axis of bar. 

**Figure 11:**
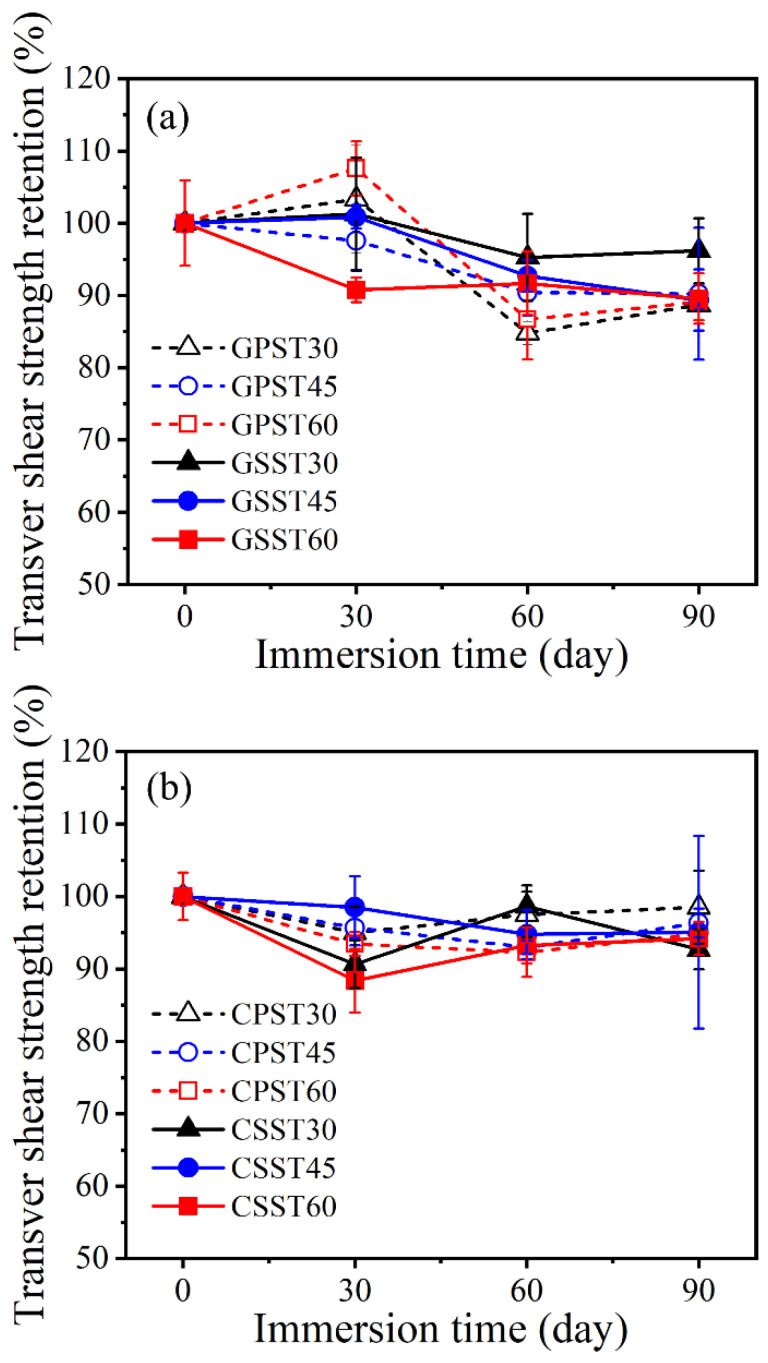
Transverse shear strength retention (**a**) GFRP and (**b**) CFRP bars.

Figure 11a,b shows the percent retained transverse shear strength of the conditioned GFRP and CFRP bars, respectively.

The transverse shear strength shows similar fluctuation with increasing exposure time as the horizontal shear strength. This phenomenon can be again ascribed to the size effect and the random variation in bar properties along its length. Therefore, the shear strength may fluctuate depending on whether the test section is weaker or stronger than the other sections located outside the test region. The strength of the different sections along the bar can vary due to differences in the cure ratio, the void content, and the nonuniformity of fiber distribution within the bar cross-section. 

### 3.5. Comparison of Mechanical Strengths Degradation

Existing experimental data [24,25,26,27,28,29,31,32,34] have shown that exposure of all types of FRP bars to the same environment generally reduces their tensile and flexural strengths more than their horizontal and transverse shear strengths. These data were mainly collected from tests involving immersion of bars in OPC concrete, or in simulated OPC pore solution, with and without sea salt. The present test results confirm these findings in the case of bars immersed in simulated CSAC pore solution, with or without sea salt. 

The horizontal shear test is not commonly used to evaluate the rate of bar degradation [70]. Although horizontal or interlaminar shear strength undergoes reduction under certain conditions, especially under prolonged high temperature exposure scenarios [4,5,27,28,29,33,38,39,41,74], it also exhibits a high degree of variability for the reasons explained earlier. One study reported a 4% increase in the horizontal shear strength of a GFRP bar after 15 years of embedment in concrete and exposure to real service conditions [75]. 

Consequently, ASTM D7705/D7705M-12 [48] does not require determination of the effect of FRP bar exposure to aggressive environments on its horizontal or transverse shear strength. It is argued here that the horizontal shear test is not necessary because it does not simulate any of the likely states of stress to which a bonded FRP reinforcing bar may be subjected in a real concrete structure. If shear lag effect in the bar is neglected, a straight reinforcing bar cross-section will be subjected to uniform normal stress only in the horizontal shear test it is put under a bending condition, where normal stress along the height of the section varies linearly, and the middle plane of the bar is subjected to maximum shear. It is difficult to envisage the latter stress state in a reinforcing bar in a reinforced concrete structure. In a bonded reinforcing bar, the change in the normal stress along the bar is equilibrated by the bond stress acting on the bar surface, so from the equilibrium point of view, there is no need for interlaminar shear stress. In an end-anchored unbonded or debonded state, the bar will be subjected to axial tension as in a conventional tension test. Neither of the latter two situations are simulated by the current horizontal shear test. 

Past research [27,28,29,33,36] has reported increased reduction of transverse shear strength with the increase in the length of immersion time. In [31], the transverse shear retention of type-C GFRP bars was reported significantly larger than 100%, whereas its tensile strength retention was around 80%. So, it is difficult to obtain a representative retained shear strength using the currently recommended test methods. The current test methods are predicated on the assumption of uniformity of the bar properties along its length; this assumption may not be always satisfied. 

As explained in [40], as a result of variations in the bar material properties and manufacturing process, each virgin bar possesses unique microstructures, including distinct voids, defects, and fiber distribution. Whereas these variations may not affect the bar tensile strength because it is determined by the strength of its weakest section along its length, the same is not true in the case of shear strengths. It is therefore suggested that the durability assessment be based on retained tensile strength only. 

### 3.6. Fourier Transform Infrared (FTIR) Spectroscopy Analysis

#### 3.6.1. Influence of Specimen Type

**Figure 12:**
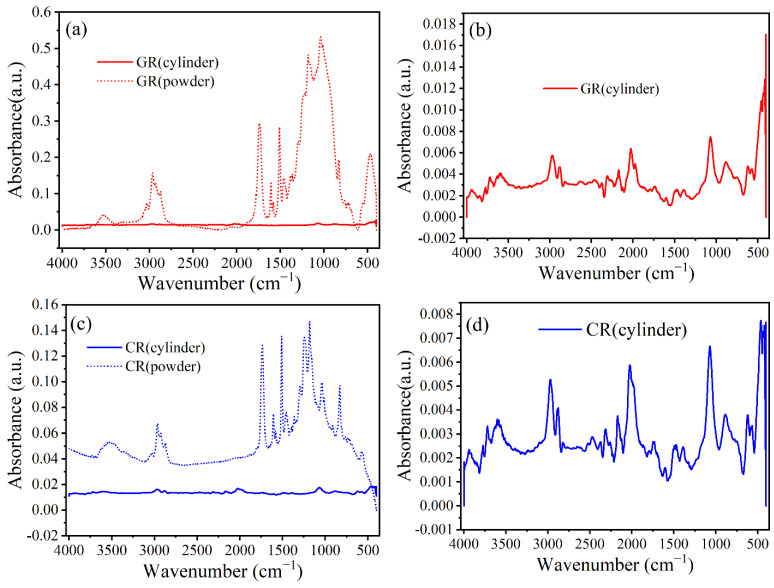
Raw FTIR results from analysis of cylinders and powder for GR and CR specimens: (**a**) cylinder and powder form of GR, (**b**) cylinder form of GR, (**c**) cylinder and powder form of CR, (**d**) cylinder form of CR.

Preliminary FTIR analysis of the matrix powder and mini-cylinders was conducted to select the suitable sample form for further analysis. The sample preparation procedures were detailed in Section 2.5. The raw results of the above analysis for the GR and CR samples are shown in Figure 12. 

A comparison of Figure 12a,c reveals that the absorbance signal of the powder for both GR and CR is significantly stronger than that of the corresponding cylinder. Although the signals of both samples exhibit clear peaks, the peaks are not the same. Since the cylinder surface could become contaminated during preparation, it was decided to use powder samples for further examination.

#### 3.6.2. FTIR Results for Powder Specimens

**Figure 13:**
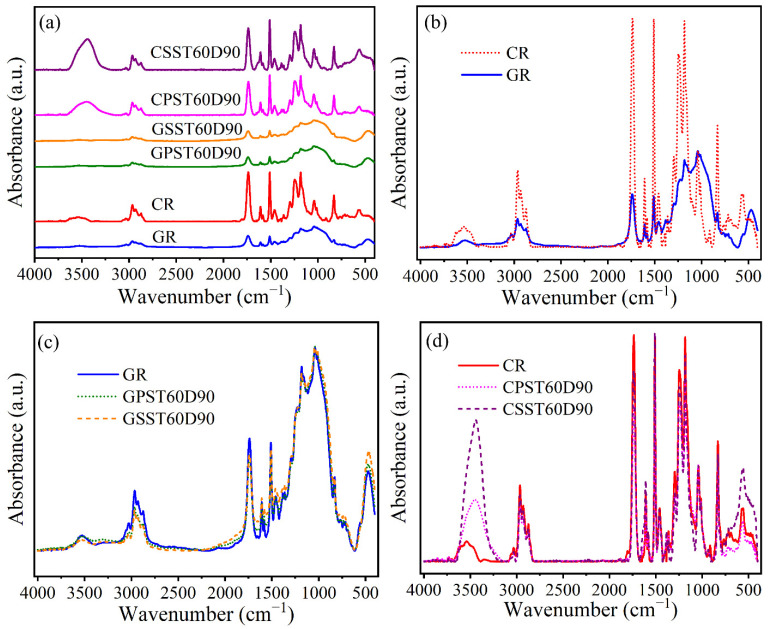
FTIR absorption spectra for the reference specimens: (**a**) reference and conditioned GFRP and CFRP, (**b**) CR and GR, (**c**) reference and conditioned GFRP, (**d**) reference and conditioned CFRP.

Figure 13 and Table 9 show the FTIR spectra and band assignments, respectively. 

As Figure 13 shows, the peak of the reference CFRP bar is significantly higher than that of the reference GFRP bar. For example, the peaks for the CFRP bar at wave numbers 1294 cm^−1^, 1236 cm^−1^ and 829 cm^−1^ are higher than those of GFRP at the same wave numbers. The two types of bars have similar patterns at wave numbers of 4000 cm^−1^~1420 cm^−1^, but at wave numbers 1420 cm^−1^~400 cm^−1^, they do not. The difference is attributed to the interference of the glass fiber in the GFRP bar. Therefore, compared to the FTIR results of the GFRP bar, the results of the CFRP bar are believed to better reveal the characteristic of the epoxy. 

To examine the post-exposure degradation of the two types of bars, the C=C bond of phenyl ring was used as the reference due to its stable chemical characteristic. Using the value of the highest peak as the representative value, the heights of O-H, C-H and C=O relative to that of C=C are shown in Table 10.

With reference to the last table, after exposure to the PS or SS solution at 60 °C for 90 days, compared to the reference GFRP bar, the relative contents of O-H, C-H and C=O in the conditioned bar exhibit obvious decrease. In the case of the conditioned CFRP, the relative content of O-H significantly increased, while the C-H and C=O in CPST60D90 and CPST60D90 generally decreased. 

As for the decrease of C=O content in the conditioned GFRP and CFRP bars, as Equation (6) reveals, OH^−^ and Cl^−^ ions will break the double bond between the oxygen and carbon atoms in the ester group, which is macroscopically reflected by increased micro-cracks and fractures [82]. Since water uptake occurs by using both the epoxy matrix and the fiber-matrix interface [83], the O-H stretching band represents the OH peaks contributed by both the cured epoxy and the absorbed water. 


(6)

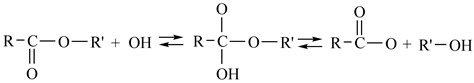



Based on the above results and their analysis, the writers believe that the reduction in the amount of C=O bonds is a better indicator of epoxy degradation than the change in the amount of OH.

### 3.7. Micromorphology and Chemical Analysis

#### 3.7.1. EDS Mapping Results

**Figure 14:**
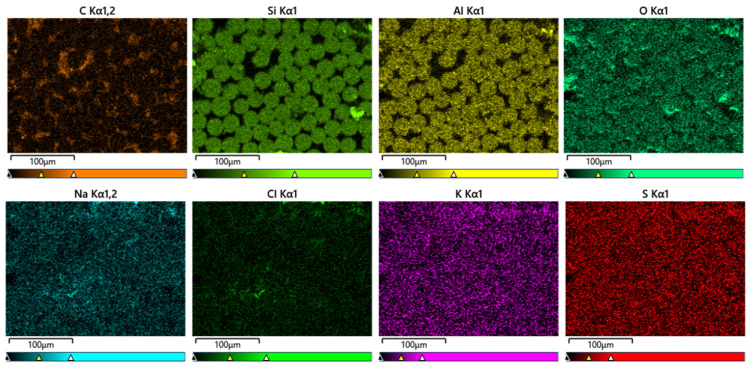
EDS mapping images of Top-1 zone in GSST60D90.

A rectangular zone approximately 280 μm by 210 μm was used for EDS mapping throughout the current SEM scanning. Figure 14 shows typical SEM images of a conditioned GFRP bar. It shows that elements Si and Al exist mainly in the zones containing glass fiber, while carbon (C) primarily exists in the matrix zone. Oxygen (O) is present in both zones. Na, Cl, K and S seem to be uniformly distributed, but their contents are small. 

**Figure 15:**
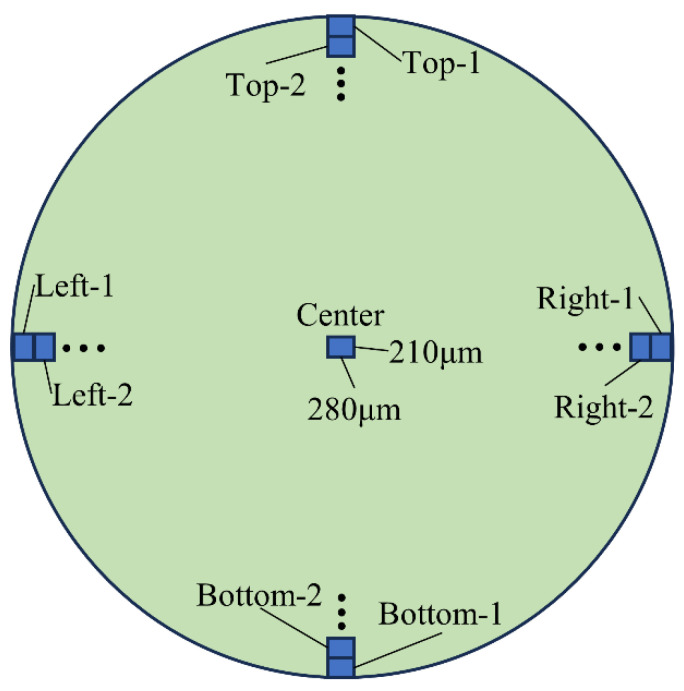
Location and labelling of EDS mapping zones, (●●● represent afterwards blue rectangular zone toward to center of specimen, detailed numbers are shown in relevant tables).

Detailed EDS results for one cross section of specimen GSST60D90 are shown in Table 11. It should be noted that the element contents in Table 11 are for the whole rectangular zone; they include the contribution of both the fibers and the matrix. By contrast, the results in Figure 14 pertain to a representative area within the rectangular zone. Figure 15 schematically identifies the location and labelling of the zones in the bar cross-section. 

Table 11 indicates the contents of Na, Cl and K ions in the four zones near the bar surface to be large, while in the zone located at the center of the cross section, they are relatively small. When the penetration depth increases, the quantity of these ions generally diminishes. Accordingly, it is reasonable to state that diffusion of Na, Cl and K ions from the solution is responsible for the higher content of these species in the zones near the GFRP bar surface. Element S, which originates from the SO_4_^2−^, has relatively high content only in zones Top-1 and Top-2. The largest depth of diffusion in zones Top, Right, Bottom and Left are about 630, 1260, 840 and 630 μm, respectively, which means that the diffusion depth is not uniform. This nonuniformity is believed to be due to the random variations in the bar cross-section microstructure and properties, a phenomenon that needs to be considered in FRP durability models that normally assume a constant diffusion depth in a bar. 

The EDS mapping results for one cross section of specimen CSST60D180 are shown in Table 12. The Na, Cl, and K ions contents in the Top-1 and Right-1 zones are significantly larger than those at the center of the cross section. Going along the radius from Top-1 towards the center, the preceding ions contents fluctuate albeit they generally decrease. On the other hand, the S element content is negligible. The largest depths of diffusion in the Top, Right, Bottom and Left directions are about 1680, 210, 210 and 210 μm, respectively, which highlights the nonuniformity of the diffusion depth.

**Figure 16:**
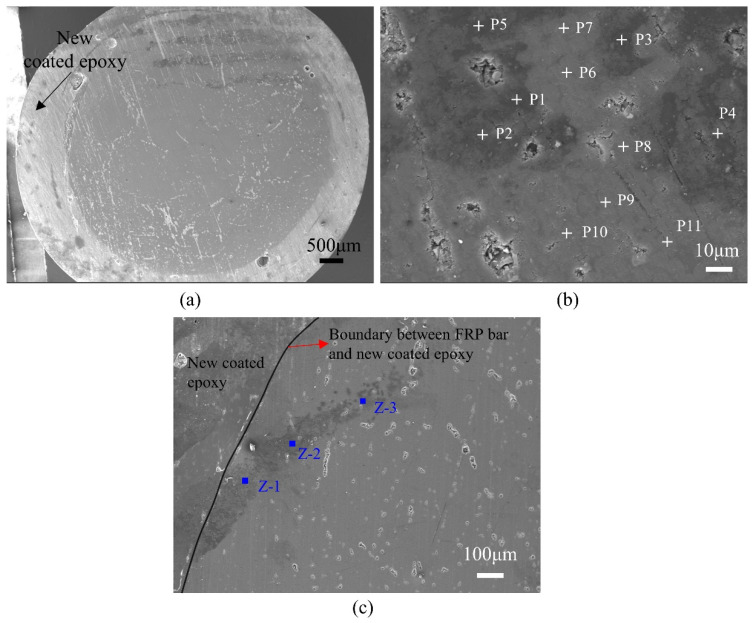
SEM images (**a**) whole cross section of CSST60D180 (**b**) Point EDS location of CSST60D180 (**c**) Overall CSST60D90.

The SEM image of an entire cross-section is shown in Figure 16a, where four distinct bandings can be observed. The clearest banding, which is believed to show the diffusion path, coincides with the zone in the top part of the bar where fluctuations in the Na, Cl and K ions contents were detected. To confirm the above assertion, additional points within the cross-section, as shown in Figure 16b, were examined, and the results are shown in Table 13. The results indicate that the Na, Cl and K ions contents in the darker regions are significantly larger than the corresponding contents in the lighter regions. This can be adduced as further evidence in support of the darker regions being diffusion paths. 

Diffusion of Na, Cl and K ions in the CFRP bar seems unusual. As Figure 16a shows, the diffusion does not occur radially as normally assumed; rather, it seems to occur along a series of horizontal secant lines. This phenomenon has not been previously reported and needs more investigation. If diffusion were to occur in this manner, then current diffusion models, which assume diffusion radially, would not be able to correctly predict the depth of the ion’s penetration. 

EDS mapping results for one cross section of CSST60D90 are shown in Table 14. The locations of Z1, Z2 and Z3 are shown in Figure 16c. As this table shows, the content of the Na, Cl and K ions decreases from Z1 to Z2 and from Z2 to Z3. Once again it points to the fact that diffusion occurred along the Z1-Z2-Z3 path, rather than radially.

#### 3.7.2. SEM Analysis of Conditioned FRP Bars

**Figure 17:**
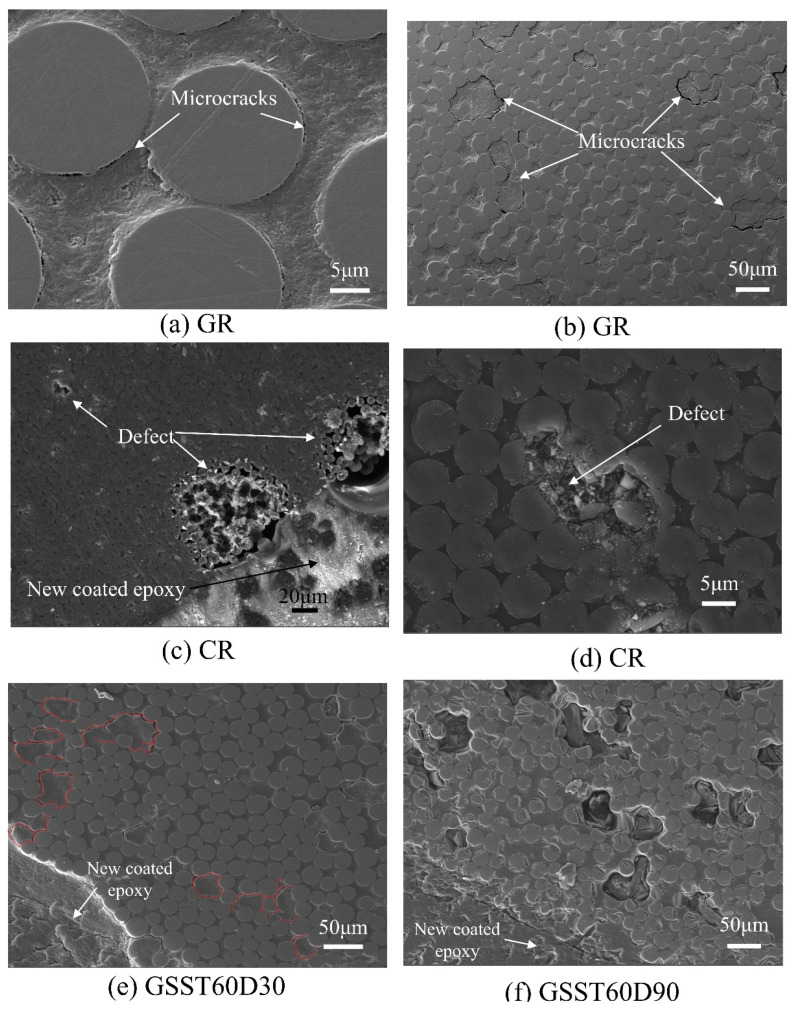

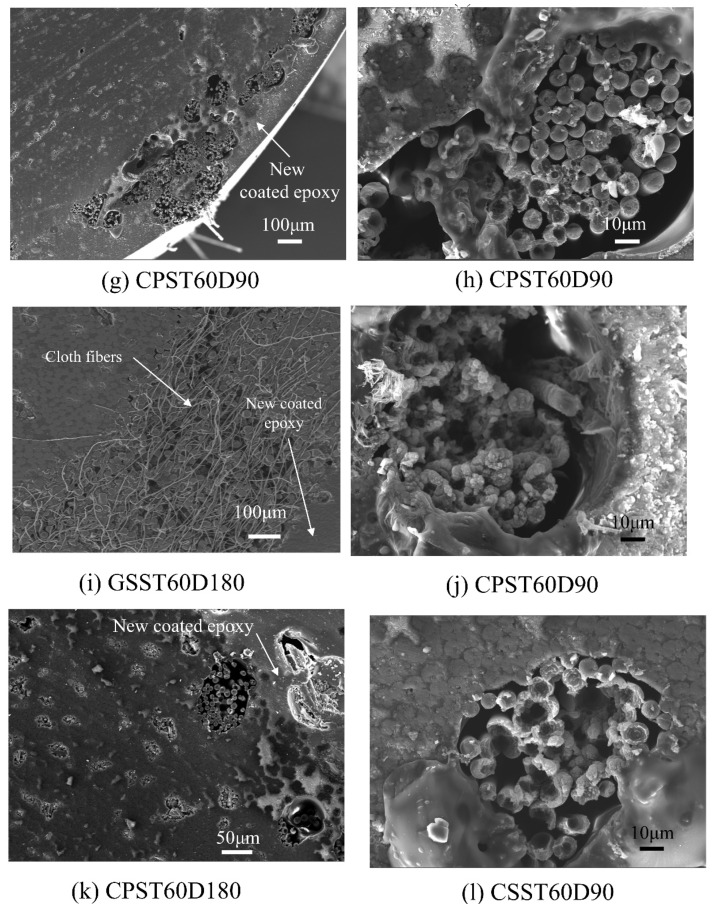
Representative micromorphology of reference and conditioned GFRP and CFRP bars: (**a**) GR, (**b**) GR, (**c**) CR, (**d**) CR, (**e**) GSST60D30, (**f**) GSST60D90, (**g**) CPST60D90, (**h**) CPST60D90, (**i**) GSST60D180 (**j**) CPST60D90, (**k**) CPST60D180, **(l**) CSST60D90.

Representative micro-morphologies of the reference and conditioned GFRP and CFRP bars are shown in Figure 17. Figure 17a,b reveals the presence of microcracks in GR. Figure 17e indicates that, after conditioning, crack density in GSST60D30 increased as new cracks formed and joined preexisting cracks. After 90 days of conditioning, severe defects, such as pits or tiny cavities (Figure 17f), formed, and the interface between the glass fibers and the epoxy matrix degraded, which is highlighted by the white lines encircling the glass fibers in Figure 17f. Figure 17i shows that the epoxy around the glass fibers in the degraded region almost vanished after 180 days of exposure. 

Figure 17c,d indicates that the interface between the carbon fibers are sound, but certain defects exist within the matrix. After 90 days of exposure to the PS solution at 60 °C, as Figure 17g shows, the number of defects at the surface of the bar increased significantly. Figure 17h shows that the epoxy in the defective zones almost totally dissolved. Figure 17j,l shows some deposits on the surface of carbon fibers, while Figure 17k shows that, after 180 days of exposure, the defects propagated toward the inner region of the CFRP bar.

#### 3.7.3. Chemical Analyses

**Figure 18:**
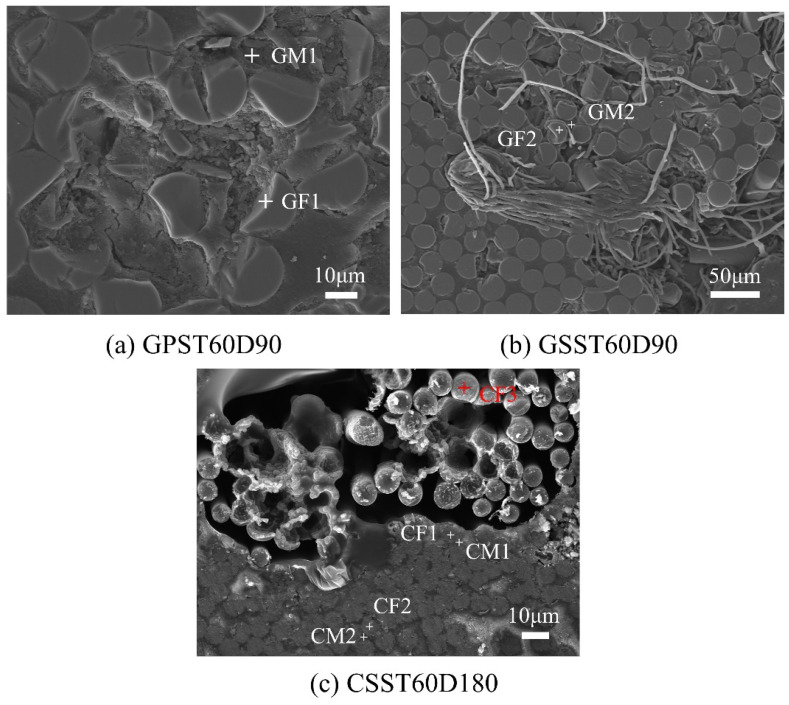
Locations of representative EDS probing points: (**a**) GPST60D90, (**b**) GSST60D90, (**c**) CSST60D180.

Figure 18 shows representative locations of the points examined by EDS in the conditioned GFRP and CFRP bars. The relevant element contents are given in Table 15. 

The data in Table 15 show that the main elements (note these may be in ionic form) in glass fiber are Si, O, Ca and Al, which broadly agrees with the EDS findings. The main element in the carbon fiber is correctly identified as C. The main elements in the GFRP matrix are identified as C, O and Zr, while those in the CFRP matrix are identified as C and O. Only around 2% Zr was found in CSST60D180. 

As for the glass fiber, the element contents at GF2, located at the fiber center, do not show an obvious change post immersion. At GF1, located near the fiber surface, Si and Ca contents show a decrease. On the other hand, the Si, Ca, Al, Na, K, S and Cl contents in the matrix at points GM1 and GM2 all show an increase to different degrees. The Si and Ca contents at GM1 exhibit dramatic increase. The increase of Si and Ca may be caused by the release of these elements due to the degradation of the glass fibers. The increase of Al, Na and K may be due to the degradation of the fibers and/or the diffusion of the solution. The increase of S and Cl could be due to the uneven distribution of S in the virgin fibers. It should be noted that diffusion of Na, Cl and K was made evident by the EDS results in Section 3.7.1. It is known that alkali ions (Na^+^, K^+^ and Ca^2+^,) will leach out of glass structure in water [82]. The bonds in Si-O-Si and Si-O-R (R=Na or K) will be broken by the hydroxyl ion and will result in the formation of soluble SiO^−^ [82].

Considering the CFRP bar, point CF3 is located in an isolated carbon fiber without being surrounded by epoxy. The elements detected at CF3 are similar to those found in the companion control bar, CR. This supports the common belief that carbon fiber is immune to attack by many chemicals, including the ones used in the current investigation, and it does not absorb water [44]. Fibers located close to the degraded zone at CF1 and CF2 also do not exibit any chemical or physical change. As for the matrix, despite the difference between the distance of points CM1 and CM2 in the epoxy from the damaged zone, they show little change in chemical composition compared to the epoxy in CR. This implies that, in the selected zone, degradation of epoxy is not widespread. 

## 4. Conclusions

GFRP and CFRP bars made with epoxy matrix were tested for their durability. The bars were 6 mm in diameter and were exposed to simulated pore solution of calcium sulfoaluminate cement concrete, with and without sea salt. The solution without salt is termed PS, and the one with salt, SS. The bars were tested for their retained tensile and horizontal and transverse shear strengths after exposure periods of 30, 60, 90 and 180 days. Each solution’s temperature was maintained at 30, 45 and 60 °C during each of the above exposure periods. The results support the following conclusions:

1. The CFRP is much less vulnerable to attack by either solution than the GFRP. Under the test conditions in this study, the maximum loss of the tensile strength was 56.9% for GFRP and 15.1% for CFRP.
2. When the solution temperature was maintained at 30 °C, irrespective of the solution type or the immersion duration, the maximum loss of the tensile strength of GFRP and CFRP did not exceed 5% and 11%, respectively.
3. Detailed microstructural and chemical analysis showed that the glass fiber, the epoxy matrix and the matrix-fiber interface all suffered damage in the GFRP bar, while in the CFRP bar, no damage was observed to the carbon fiber.
4. The presence of sea salt significantly increased the degradation of the GFRP bar, but its effect on the CFRP was relatively small.
5. Increase of PS temperature from 45 to 60 °C reduced the retained tensile strength (RTS) of the GFRP bar from 90.7% to 59.3% after 180 days of exposure. The corresponding values were 78.9% and 43.1% for the same bar immersed in the SS solution.
6. The RTS of the GFRP bar dropped from 83.0% to 59.3% when the exposure duration in the PS solution maintained at 60 °C was increased from 90 to 180 days. The corresponding values for immersion in the SS solution under identical conditions were 73.6% and 43.1%.
7. Increase of PS temperature from 45 to 60 °C reduced the RTS of the CFRP bar from 90.8% to 87.0% after 180 days of exposure. The corresponding values were 90.7% and 84.9% for the same bar immersed in the SS solution.
8. The RTS of the CFRP dropped from 98.0% to 87.0% when the exposure duration in the PS solution maintained at 60 °C was increased from 90 to 180 days. The corresponding RTS for immersion in the SS solution under identical conditions dropped from 97.0% to 84.9%.
9. The degradation of the horizontal and transverse shear strengths compared to that of the tensile strength was generally smaller under the same exposure conditions. However, due to the likely nonuniformity of each type of bar properties along its length, the results were inconsistent. Therefore, the current results show that, unless the bar is produced under stringent quality control conditions, its durability cannot be assessed through its %retained shear strength.
10. Based on the relevant ASTM standard, the CFRP bar satisfies the alkali resistance requirement of the standard in the CSAC pore solution with and without salt, whereas the GFRP bar satisfies this requirement in the PS solution but not in the SS.

The present work is an initial investigation, and the results are based on the testing of bars from a single manufacturer. To ascertain the generality of the current results, similar tests on FRP bars from different manufactures need to be performed. 

## Figures and Tables

**Table 1:** Physical and mechanical properties of reference GFRP and CFRP bars.

Property Type	Property	Relevant ASTM Standard	GFRP		CFRP	
			Mean Value	COV%	Mean Value	COV%
Physical	Nominal cross-sectional area, mm^2^	D792-20 [54]	26.6	1.1	26.5	1.7
	Effective bar diameter, mm	D7205/D7205M-21 [51]	5.8	0.5	5.8	0.9
	Fiber content, wt%	D2584-18 [55]	82.9	0.3	32.5	6.1
	Glass transition temperature, °C	E1356-08 (Reapproved 2014) [56]	104.0	-	92.1	-
Mechanical	Ultimate tensile strength, MPa	D7205/D7205M-21 [51]	1223	2.6	1902	6.2
	Tensile modulus of elasticity, GPa	D7205/D7205M-21 [51]	53.4	1.9	147.3	1.4
	Horizontal shear strength, MPa	D4475-21 [49]	52.8	3.7	50.9	5.6
	Transverse shear strength, MPa	D7617/D7617M-11 (Reapproved 2017) [50]	257.7	5.9	293.3	4.5

**Table 2:** Chemical compositions of simulated pore solutions in this study.

Type	Quantities (Gram per Liter)						pH (at 25 °C)
	Simulated CSAC Pore Solution [11]			Simulated Seawater Composition [57]			
	KOH	NaAlO_2_	K_2_SO_4_	NaCl	Na_2_SO_4_	KCl	
PS	1.403	9.017	9.584	-	-	-	12.9
SS	2.132	9.017	9.584	32.136	4.09	0.695	12.9

**Table 3:** Tensile and elastic modulus of control and conditioned GFRP bars.

Specimen	Tensile Strength			Elastic Modulus	
	Mean (MPa)	% Retained	COV (%)	Mean (GPa)	COV (%)
GR (Control)	1223	100.0	2.63	53.42	1.91
GPST30D30	1194	97.7	3.74	54.80	3.48
GPST30D60	1207	98.6	3.97	52.98	0.80
GPST30D90	1187	97.0	3.82	53.9	2.73
GPST30D180	1170	95.7	5.41	53.7	1.66
GPST45D30	1199	98.1	3.95	53.5	3.38
GPST45D60	1200	98.1	1.83	52.1	3.61
GPST45D90	1192	97.5	2.14	53.1	1.69
GPST45D180	1109	90.7	6.50	54.5	2.79
GPST60D30	1169	95.6	2.95	54.1	2.95
GPST60D60	1127	92.2	3.65	51.9	1.16
GPST60D90	1016	83.0	5.40	51.6	1.60
GPST60D180	725	59.3	14.69	53.0	1.38
GSST30D30	1223	100.0	3.42	52.4	3.53
GSST30D60	1190	97.3	1.89	52.7	1.35
GSST30D90	1185	96.9	1.60	52.9	3.97
GSST30D180	1173	95.9	2.37	53.5	3.70
GSST45D30	1212	99.1	2.62	52.4	2.98
GSST45D60	1183	96.8	3.65	53.1	1.74
GSST45D90	1167	95.4	0.88	52.2	2.18
GSST45D180	965	78.9	12.37	53.4	3.44
GSST60D30	1158	94.7	5.37	52.3	1.35
GSST60D60	1043	85.3	5.65	52.6	3.03
GSST60D90	900	73.6	12.67	52.3	1.99
GSST60D180	527	43.1	5.00	51.7	1.99

**Table 4:** Tensile and elastic modulus of control and conditioned CFRP bars.

Specimen	Tensile Strength			Elastic Modulus	
	Mean (MPa)	% Retained	COV (%)	Mean (MPa)	% Retained
CR	1902	100.0	6.15	147.26	1.35
CPST30D30	1919	100.9	2.11	149.88	2.30
CPST30D60	1866	98.1	2.37	149.86	1.76
CPST30D90	1879	98.8	1.95	150.85	0.47
CPST30D180	1783	93.7	1.60	143.32	1.26
CPST45D30	1940	102.0	3.33	151.21	1.70
CPST45D60	1899	99.8	3.49	150.44	1.52
CPST45D90	1915	100.7	2.71	146.15	1.65
CPST45D180	1727	90.8	3.33	145.58	0.52
CPST60D30	1893	99.5	3.98	150.08	2.11
CPST60D60	1884	99.0	6.01	150.53	1.35
CPST60D90	1864	98.0	4.78	146.61	1.95
CPST60D180	1655	87.0	9.85	139.43	4.27
CSST30D30	1869	98.2	3.84	150.46	0.63
CSST30D60	1924	101.1	2.33	151.15	0.53
CSST30D90	1919	100.9	2.55	151.11	0.88
CSST30D180	1707	89.7	7.25	141.65	3.65
CSST45D30	1867	98.2	2.95	147.95	1.57
CSST45D60	1897	99.7	5.20	151.75	1.74
CSST45D90	1847	97.1	3.36	143.99	2.33
CSST45D180	1726	90.7	6.61	144.70	1.51
CSST60D30	1847	97.1	4.14	148.00	1.34
CSST60D60	1952	102.6	2.83	150.44	0.71
CSST60D90	1845	97.0	3.93	148.24	1.80
CSST60D180	1615	84.9	7.37	137.58	5.22

**Table 5:** Significance of pore solution influence on bars retained tensile strength.

Sample	GT45D180	GT60D60	GT60D180
*p*-value	0.049	0.025	0.004

**Table 6:** Statistical analysis of test data for determining the significance of temperature effect on the retained tesile strength of the test bars.

Sample	Y/N	Significance of Post-Mortem Comparison between Different Immersion Temperature	Notes
GPSD30	N ^a^	0.457	
GPSD60	Y ^a^	0.005(30,60), 0.007(45,60)	
GPSD90	N	0.816(30,45)	*t*-test ^b^, GPST60D90 is not contained
GPSD180	Y	<0.001(30,60), <0.001(45,60)	
GSSD30	N	0.066	
GSSD60	Y	<0.001 (30, 60), <0.001(45,60)	
GSSD90	Y	<0.001 (30,60)	*t*-test, GSST45D90 is not contained
GSSD180	Y	0.001(30,45), <0.001(30,60), <0.001(45,60)	

Note: ^a^ Y = yes significant; N = not significant at 95% confidence level; ^b^ *t*-test: independent sample *t*-test was used because data not satisfied homogeneity of variance or data only contained only 2 levels.

**Table 7:** Notability analysis results of immersion time.

Sample	Y/N	Significance of Post-Mortem Comparison between Different Immersion Days	Notes
GPST30	N ^a^	0.711	
GPST45	Y ^a^	0.005(30,180), 0.003(60,180), 0.007(90,180)	
GPST60	Y	<0.001(30,180), <0.001(60,180)	GPST60CD90 is not included
GSST30	N	0.051	
GSST45	Y	<0.001(30,180), <0.001(60,180)	GSST45D90 is not included
GSST60	Y	0.015(30,60), <0.001(30,90), <0.001(30,180), 0.003(60, 90), <0.001(660, 180), <0.001(90, 180)	
CPST30	Y	0.034(30,60),0.000(30,180),0.002(60,180),0.001(90,180)	
CPST45	Y	0.000(30,180), 0.000(60,180), 0.000(90,180)	
CPST60	Y	0.002(30,180), 0.003(60,180),0.005(90,180)	
CSST30	Y	0.020(30,180),0.016(60,90),0.007(90,180)	*t*-test ^b^
CSST45	Y	0.012(30,180), 0.003(60,180), 0.029(90,180)	
CSST60	Y	0.043(30,60), 0.039(60,90),0.000(30,180), 0.000(60,180), 0.000(90,180)	

Note: ^a^ Y = yes significant; N = not significant at 95% confidence level; ^b^ *t*-test: independent sample *t*-test was used because data not satisfied homogeneity of variance or data only contained only 2 levels.

**Table 8:** *p*-value of Statistical analyses.

Type	Shapiro–Wilk			Levene’s Test	One-Way ANOVA
	225	150	100		
GFRP bars	0.207	0.386	0.331	0.774	0.573
CFRP bars	0.942	0.661	0.718	0.572	0.558

**Table 9:** Assignments of the main characteristic absorption bands.

Functional Group	Assignment of Wave Numbers to Groups in the Bars Examined					
	GR	CR	GPS T60D90	GSS T60D90	CPS T60D90	CSS T60D90
O-H stretching [76,77]	3524	3540	3524	3536	3449	3438
C-H stretching C-H from phenyl ring [76,77]	3028	3033	3031	3029	3033	3033
C-H stretching from alkyl [76,77]	2965, 2931, 2874	2964, 2929, 2873	2965, 2931, 2874	2965, 2931, 2874	2964, 2930, 2873	2964, 2931, 2873
C=O stretching in a non-conjugate ester group [78]	1739	1736	1739	1739	1735	1736
C=C stretching in phenyl ring [76,77,79,80]	1608, 1510	1607, 1510	1608, 1510	1608, 1510	1607, 1509	1608, 1510
C-O [76]	1294, 1228	1295, 1246	1295, 1238	1291, 1234	1295, 1247	1295, 1246
C-O aromatic ring stretching [5]+ stretching vibration of Si-O-Si [81]	1182	1183	1182	1182	1182	1182
Stretching vibration of C-O-φ [5,78] + stretching vibration of Si-O-Si [81]	1040	1040	1040	1040	1042	1041
C-H bending in benzene ring [5,77]	829	829	829	829	829	829

**Table 10:** Relative height of representative bonds.

Type	OH (3438–3540)	CH (2965)	C=O (1739)	C=C (1510)
GR	0.144	0.557	1.038	1
GPST60D90	0.138	0.436	0.952	1
GSST30D90	0.108	0.402	0.950	1
CR	0.090	0.337	1.002	1
CPST60D90	0.334	0.314	0.837	1
CSST60D90	0.617	0.289	0.835	1

**Table 11:** EDS mapping results of one cross section of GSST60D90.

GSST60D90	Depth (μm)	C	O	Na	Al	Si	S	Cl	K	In Total
Center		36.83	27.28	0.58	6.32	28.00	0.81	0.08	0.10	100
Top-1	0–210	32.37	24.92	1.87	6.39	31.04	1.24	1.63	0.53	100
Top-2	210–420	34.69	24.56	0.72	6.40	31.74	1.06	0.51	0.31	100
Top-3	420–630	36.02	25.20	0.51	6.45	30.54	0.98	0.13	0.17	100
Right-1	0–210	39.91	25.80	1.61	5.32	24.46	1.01	1.44	0.45	100
Right-2	210–420	40.40	25.46	1.11	5.49	25.37	0.98	0.87	0.32	100
Right-3	420–630	39.95	25.25	0.79	5.78	26.65	0.92	0.40	0.27	100
Right-4	630–840	37.57	25.98	0.75	6.12	28.08	0.91	0.37	0.23	100
Right-5	840–1050	39.42	25.70	0.66	5.94	26.94	0.89	0.26	0.20	100
Right-6	1050–1260	39.10	26.16	0.58	6.37	26.69	0.81	0.10	0.18	100
Bottom-1	0–210	41.61	27.04	1.00	5.13	23.56	0.83	0.51	0.31	100
Bottom-2	210–420	42.07	27.15	1.16	5.16	22.62	0.83	0.74	0.27	100
Bottom-3	420–630	41.25	27.39	0.61	5.47	24.18	0.72	0.22	0.15	100
Bottom-4	630–840	42.57	27.31	0.44	6.37	22.33	0.75	0.10	0.12	100
Left-1	0–210	43.00	26.72	1.78	4.61	20.82	0.81	1.96	0.31	100
Left-2	210–420	39.60	27.30	1.11	5.49	24.70	0.77	0.91	0.11	100
Left-3	420–630	40.20	27.66	0.46	5.81	24.85	0.78	0.10	0.14	100

**Table 12:** EDS mapping results of one cross section of CSST60D180.

CSST60D180	Depth	C	O	Na	Al	Si	S	Cl	K	In Total
Center		87.32	11.86	0.13	0.00	0.24	0.30	0.08	0.06	100
Top-1	0–210	73.06	22.26	1.01	0.06	1.55	0.43	1.31	0.32	100
Top-2	210–420	77.75	19.02	0.63	0.01	1.38	0.46	0.51	0.24	100
Top-3	420–630	80.09	16.49	0.58	0.10	1.81	0.38	0.35	0.21	100
Top-4	630–840	79.49	16.53	0.76	0.04	2.00	0.43	0.52	0.23	100
Top-5	840–1050	82.30	15.14	0.37	0.01	1.61	0.40	0.14	0.04	100
Top-6	1050–1260	80.51	15.77	0.87	0.03	1.72	0.43	0.42	0.25	100
Top-7	1260–1470	82.29	14.61	0.67	0.02	1.50	0.36	0.36	0.19	100
Top-8	1470–1680	85.51	13.15	0.10	0.00	0.69	0.35	0.14	0.06	100
Right-1	0–210	80.28	17.85	0.38	0.00	0.70	0.38	0.24	0.16	100
Right-2	210–420	83.68	13.79	0.15	0.13	1.83	0.34	0.09	0.00	100
Right-3	420–630	83.24	14.36	0.20	0.12	1.65	0.34	0.06	0.03	100
Bottom-1	0–210	81.89	15.89	0.20	0.12	1.42	0.33	0.14	0.02	100
Bottom-2	210–420	81.78	15.27	0.16	0.53	1.82	0.31	0.11	0.02	100
Left-1	0–210	82.00	16.30	0.09	0.00	1.08	0.39	0.10	0.04	100
Left-2	210–420	84.39	14.27	0.01	0.00	0.91	0.37	0.00	0.04	100

**Table 13:** EDS Point results for CSST60D180.

Shade	Label	C	O	Na	Al	Si	S	Cl	K	In Total
Darker	p1	81.06	15.32	1.70	0.00	0.12	0.84	0.55	0.41	100
Darker	p2	85.79	11.23	1.44	0.00	0.23	0.40	0.49	0.42	100
Darker	p3	80.11	14.30	2.93	0.00	0.34	0.49	1.09	0.74	100
Darker	p4	86.92	9.67	1.48	0.00	0.38	0.53	0.62	0.41	100
Darker	p5	85.72	11.08	1.48	0.00	0.12	0.47	0.70	0.43	100
Lighter	p6	94.43	4.83	0.24	0.00	0.07	0.36	0.08	0.00	100
Lighter	p7	89.54	9.13	0.17	0.00	0.60	0.37	0.14	0.05	100
Lighter	p8	91.50	4.68	0.36	0.21	2.80	0.38	0.07	0.00	100
Lighter	p9	94.76	4.36	0.24	0.00	0.15	0.37	0.11	0.02	100
Lighter	p10	92.90	5.98	0.22	0.00	0.28	0.49	0.05	0.07	100
Lighter	p11	84.45	13.22	0.15	0.00	0.35	1.05	0.71	0.07	100

**Table 14:** EDS mapping results of CSST60D90.

CPST60D90	C	O	Na	Al	Si	S	Cl	K	In Total
Z1	86.48	11.23	0.86	0.00	0.58	0.25	0.41	0.20	100
Z2	88.07	10.26	0.60	0.02	0.33	0.21	0.33	0.18	100
Z3	88.36	8.44	0.33	0.00	2.30	0.22	0.24	0.10	100

**Table 15:** EDS test results of representative GFRP and CFRP specimens.

Specimen Label	Type	Spectrum	Percent of Chemical Elements in Weight (%)													
			C	O	Na	Mg	Al	Si	S	Cl	K	Ca	Ti	Fe	Zr	Total
GR	fiber		2.29	37.41	0.36	1.54	6.87	27.63	0.22	0.03	0.24	15.95	0.21	0.18	7.05	100
GR	matrix		68.08	15.64	0.10	0.27	0.32	1.48	0.66	0.46	0.00	0.94	0.04	0.22	11.79	100
GPST60D90	matrix	GM1	40.02	16.52	0.30	0.52	2.70	12.65	1.89	0.60	0.71	11.49	0.22	0.97	11.42	100
GPST60D90	fiber	GF1	28.18	28.26	0.31	0.95	4.52	18.45	0.26	0.04	0.20	11.28	0.17	0.23	7.15	100
GSST60D90	matrix	GM2	68.01	7.59	0.34	0.13	0.60	1.80	1.24	0.76	0.34	1.83	0.05	0.07	17.24	100
GSST60D90	fiber	GF2	4.84	36.74	0.37	1.53	6.63	26.48	0.18	0.06	0.25	15.63	0.25	0.18	6.86	100
CR	fiber		99.12	0.78	0	0.02	0.01	0.01	0.01	0.01	0	0	0.02	0	0.02	100
CR	matrix		94.72	4.54	0.02	0.01	0.01	0.05	0.03	0.42	0	0	0.03	0	0.16	100
CR	matrix		88.17	11.14	0	0	0.07	0.05	0.05	0.35	0.11	0	0.03	0.03	0	100
CSST60D180	fiber	CF3	98.56	0.93	0	0	0	0.02	0.01	0.01	0	0	0	0.02	0.45	100
CSST60D180	fiber	CF1	97.69	1.54	0.18	0.01	0	0.05	0.02	0.01	0	0.02	0.02	0	0.45	100
CSST60D180	matrix	CM1	90.1	6.88	0.07	0.01	0.02	0.27	0.06	0.31	0	0.13	0	0	2.15	100
CSST60D180	fiber	CF2	97.25	2.17	0.11	0	0.02	0.03	0.02	0.01	0.02	0	0.01	0	0.37	100
CSST60D180	matrix	CM2	90.05	6.77	0.05	0	0.03	0.02	0.18	0.19	0.02	0.1	0.07	0.26	2.27	100

## Data Availability

Data will be made available on request.
